# Case reports: chemoradiotherapy for locally advanced neuroendocrine carcinoma of the gallbladder

**DOI:** 10.1007/s12328-022-01645-1

**Published:** 2022-06-02

**Authors:** Yuma Takeda, Noritoshi Kobayashi, Takaomi Kessoku, Naoki Okubo, Akihiro Suzuki, Motohiko Tokuhisa, Haruo Miwa, Naoko Udaka, Yasushi Ichikawa

**Affiliations:** 1 Department of Oncology, Yokohama City University School of Medicine Graduate School of Medicine, Yokohama, Japan; 2 Department of Palliative Medicine, Yokohama City University Hospital, Yokohama, Japan; 3 Department of Gastroenterology and Hepatology, Yokohama City University School of Medicine Graduate School of Medicine, Yokohama, Japan; 4 Gastroenterological Center, Yokohama City University Medical Center, Yokohama, Japan; 5 Department of Pathology, Yokohama City University Hospital, 3-9, Fuku-ura, Kanazawa-ku, Yokohama, 2360004 Japan

**Keywords:** Neuroendocrine tumor, Neuroendocrine carcinoma, Gallbladder, Chemoradiotherapy, Complete response

## Abstract

Neuroendocrine carcinoma (NEC) is a rare subtype of malignant gallbladder tumor. Although surgical resection is the only potentially curative therapy for gallbladder NEC, most cases are surgically unresectable because of advanced stage disease and/or biologically aggressive behavior. The standard palliative treatment for malignant gallbladder tumors is chemotherapy; however, the efficacy of chemoradiotherapy in the treatment of gallbladder tumors is controversial. Here, we report a case of gallbladder NEC that showed a durable response to chemoradiotherapy. A 68-year-old Japanese man presented with a huge gallbladder tumor with liver and duodenal invasion. Pathological findings revealed poorly differentiated NEC of the gallbladder. After seven cycles of chemotherapy comprising cisplatin and irinotecan, computed tomography (CT) revealed remarkable tumor shrinkage, but an enlarged portal lymph node. The patient was treated with 50.4 Gy in 28 fractions with two cycles of cisplatin and etoposide. After chemoradiotherapy, the enlarged lymph node also decreased in size. Maximum standardized uptake value of fluorodeoxyglucose-positron emission tomography/CT(FDG-PET/CT) changed from 8.2 to physiological accumulation. We defined this condition as a complete response on both enhanced CT and FDG-PET/CT; therefore, we did not perform systemic treatment and only observed his condition. This patient remained healthy with no recurrence at 3 years after chemoradiotherapy.

## Introduction

Neuroendocrine neoplasms (NENs) originate from neuroendocrine cells located throughout the whole body, most commonly in the pancreas, lung, and gastrointestinal tract [1, 2].

NENs are generally subclassified by primary lesion and pathological findings, including tumor differentiation and grade. Neuroendocrine carcinoma (NEC) is defined as a poorly differentiated and high-grade tumor. NEC is diagnosed upon pathological examination with immunohistochemical staining according to the 2019 World Health Organization (WHO) classification of tumors of the digestive system [3].

NEC is a rare subtype of gallbladder tumor that accounts for 0.5% of all NENs and 2% of all gallbladder tumors [2]. Almost all patients with NEC of the gallbladder are diagnosed incidentally based on pathological examination, including immunohistochemical findings [4]. The clinicopathological characteristics and standard treatment strategy for NEC of the gallbladder remains undetermined due to its rarity. Further, although surgical resection is the only potentially curative therapy for gallbladder NEC, most cases cannot be surgically resected because of advanced stage disease and/or locally aggressive behavior. The standard palliative treatment for malignant gallbladder tumors is chemotherapy; however, the efficacy of chemoradiotherapy in the treatment of gallbladder tumors is controversial. We herein describe a case of gallbladder NEC in which the patient underwent chemoradiotherapy and showed a complete response to chemoradiotherapy.

## Case report

**Fig. 1:**
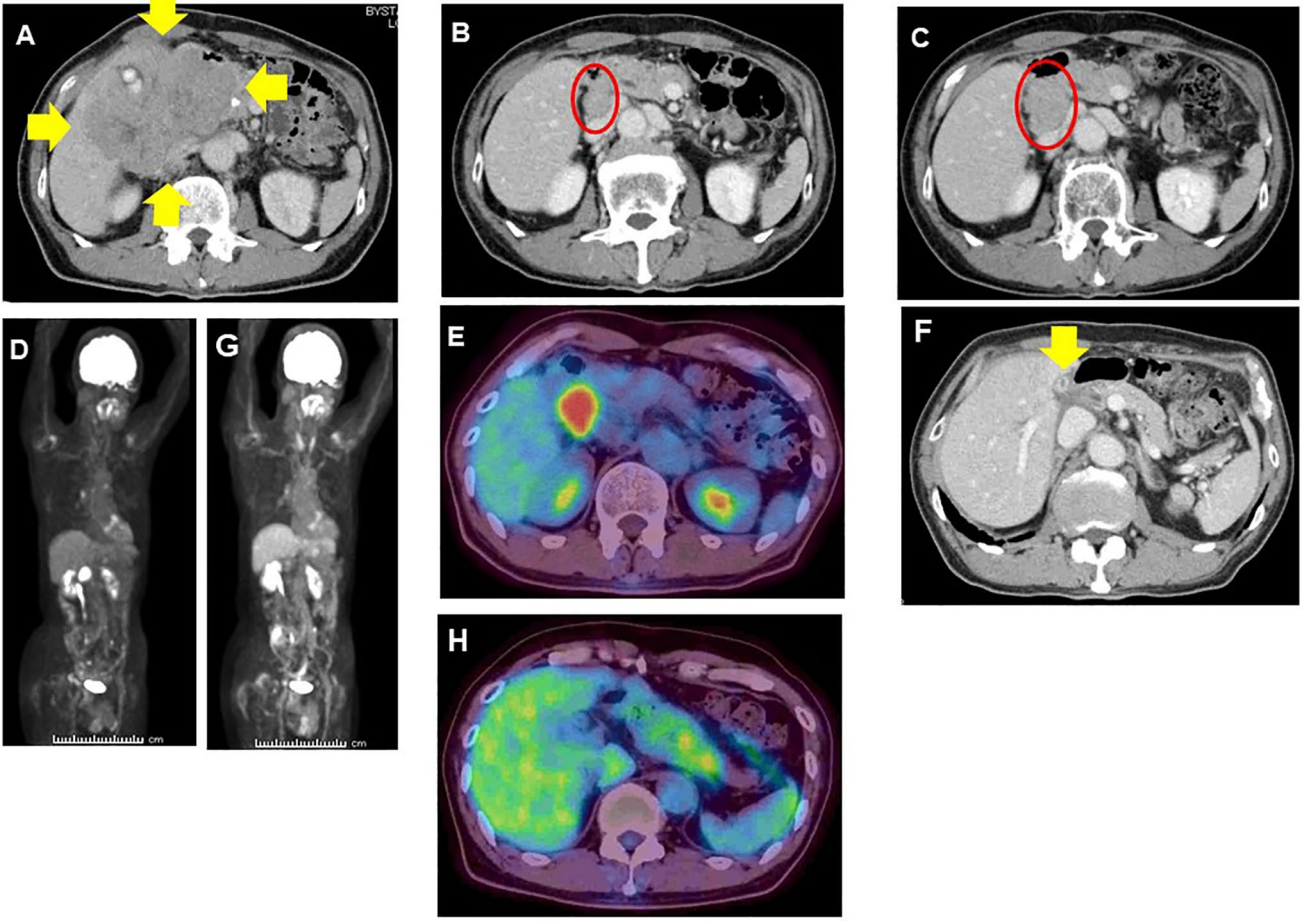
Enhanced computed tomography (CT) and fluorodeoxyglucose-positron emission tomography-computed tomography (FDG-PET/CT) images. Enhanced CT showing a gallbladder mass before treatment (**a**). The tumor size was about 130 mm. Two high-density spots revealed gallbladder stones (yellow arrowhead) and a high-density dot was bile duct plastic stent for biliary drainage (yellow arrow). Two cycles of chemotherapy resulted in a partial response with remarkable tumor shrinkage (**b**). After four cycles chemotherapy, tumor response was continuing (**c**). After five cycles of chemotherapy, the primary huge tumor lesions disappeared and distal migration of the plastic stent (**d**). However, the hepatic lymph node surrounding the hepatic hilum was slightly enlarged, about 18 mm (yellow arrowhead). After seven cycles of chemotherapy, the lymph node was remarkably enlarged, about 28 mm (**e**) (yellow arrowhead). Maximum intensity projection (MIP) (**f**) and FDG-PET/CT imaging (**g**) before chemoradiotherapy revealed uptake in the portal lymph node (SUV max 8.2) (yellow arrowhead). After chemoradiotherapy, the enlarged hepatic lymph node shrinked, and the CT value of panniculitis around the gallbladder slightly increased (**h**) (yellow arrowhead). MIP (**i**) and FDG-PET/CT imaging (**j**) after chemoradiotherapy did not reveal increased uptake in the gallbladder and lymph node

A 68-year-old man with no comorbidities presented to a local hospital with worsening back pain for two weeks. Contrast computed tomography (CT) of the abdomen and pelvis showed a huge mass of 130 mm with liver and duodenal invasion (Fig. 1a). There was no evidence of a mass or metastatic disease in the chest, bone, or brain.

**Fig. 2:**
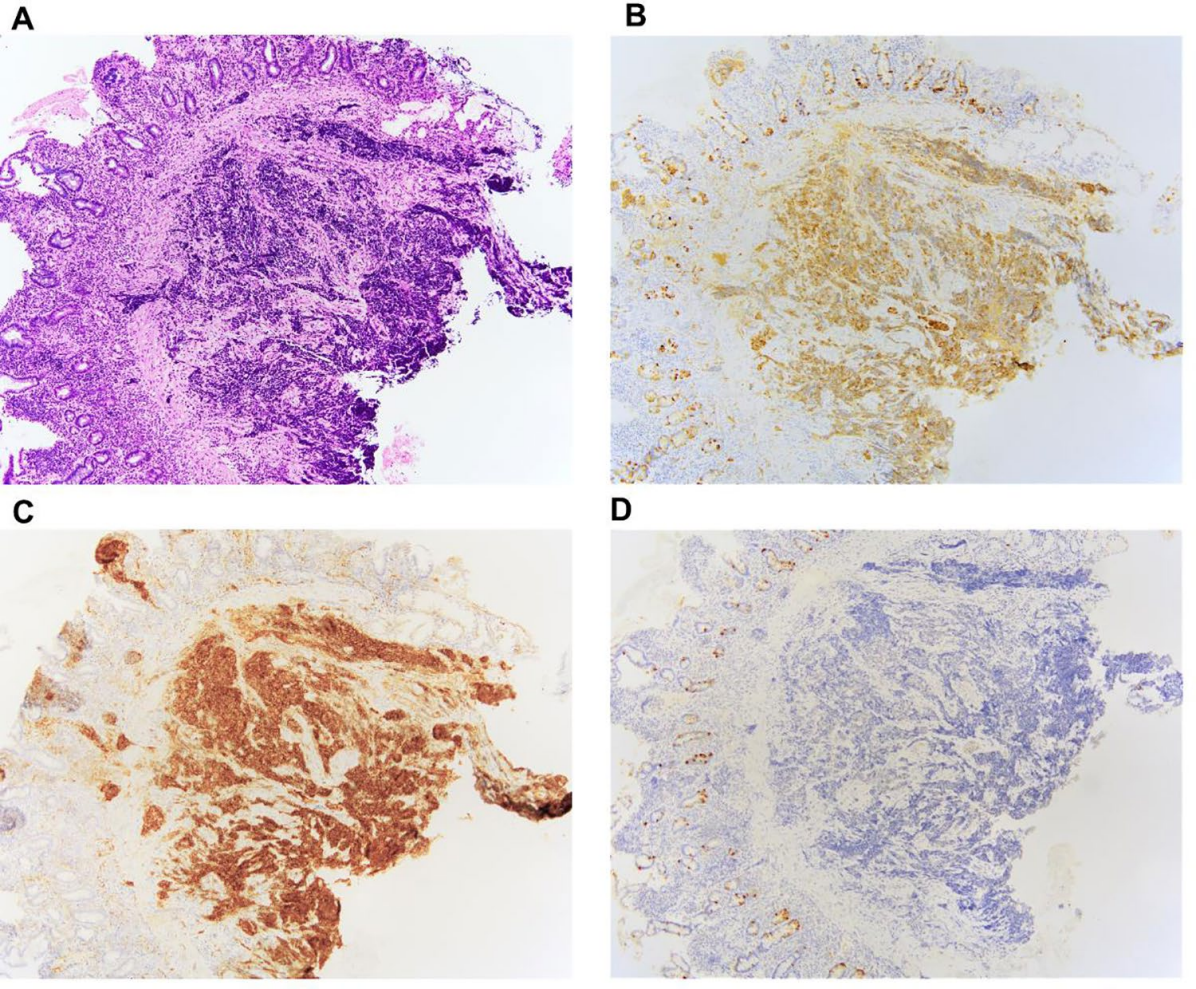
Microscopic findings of duodenal and liver biopsy specimens. Hematoxylin–eosin staining revealed small tumor cells arranged in nests and sheets in a sub-epithelial lesion (**a**). Tumor cells were small and round in shape with minimal cytoplasm and prominent nuclei (original magnification × 400). Immunohistological examinations showed synaptophysin (**b**) and CD56 (**c**) immune positivity, but chromogranin A immune negativity (original magnification × 400). Ki-67 Labeling index was 68.1% (**d**)

Subsequently, the endoscopic duodenal and liver biopsy specimens were indicative of proliferation of small round cells with high nuclear-to-cytoplasmic ratios and a poorly differentiated adenocarcinoma (Fig. 2a). Thereafter, immunohistochemical staining revealed that the tumor cells were diffuse positive for synaptophysin and CD56 and negative for chromogranin A (Fig. 2b, c). The Ki-67 labeling index of the tumor from the liver specimen was 68.1% (Fig. 2d). Based on pathological and imaging findings, a final diagnosis of neuroendocrine carcinoma (small cell type) of gallbladder origin with liver and duodenal invasion was made. The tumor was deemed unresectable due to its very huge size and because it was locally advanced with invasion of the surrounding organs. Therefore, palliative chemotherapy was administered.

The patient received five cycles of chemotherapy consisting of cisplatin and irinotecan. CT findings revealed shrinkage of the primary gallbladder tumor (Fig. 1b and c). However, slight enlargement of the hepatic hilar lymph node was observed (Fig. 1d). After an additional two cycles of chemotherapy, CT revealed apparent hepatic lymph node swelling (max 28 mm) (Fig. 1e). Fluorodeoxyglucose-positron emission tomography/CT(FDG-PET/CT) revealed high uptake in this lesion (Maximum standardized uptake value, SUV max 8.2) (Fig. 1f, g). This is considered a progressive disease associated with chemotherapy. He was then referred to our hospital for further treatment. The physical examination results were unremarkable. He underwent routine blood tests, which revealed no remarkable changes, including tumor markers (Table1).

**Table 1:** Laboratory findings before chemoradiotherapy

Serum parameter	Normal range	Before chemoradiotherapy
WBC (/µL)	3.3–8.6	4700
Hb (g/dL)	13.7–16.8	11.6
PLT (× 10^4^/μL)	158–348	145
AST (IU/L)	13–30	22
ALT (IU/L)	10–42	18
T-Bil (mg/dL)	0.4–1.5	0.5
Cre (mg/dL)	0.65–1.07	1.04
TP (g/dL)	6.6–8.1	6.9
Alb (g/dL)	4.1–5.1	4.1
CRP (mg/dL)	< 0.14	0.04
CEA (ng/mL)	0.6–3.8	3.2
CA19-9 (U/mL)	1–29	1
NSE (ng/mL)	< 16.3	9.0
Pro GRP (pg/mL)	< 81	56.7

*WBC* white blood cells, *Hb* hemoglobin, *PLT* platelet test, *AST* aspartate aminotransferase, *ALT* alanine aminotransferase, *T-Bil* bilirubin test, *Cre* creatinine, *TP* total protein, *Alb* albumin, *CRP* C-reactive protein, *CEA* carcinoembryonic antigen, *CA19-9* carbohydrate antigen 19-9, *NSE* neuron specific enolase, *pro GRP* pro-gastrin-releasing peptide

At first, we considered and recommended surgical resection, but the patient refused our proposal. He had already received first-line chemotherapy. However, the evidence for second-line chemotherapy is not established, and it is very important to control local progression in this case. Therefore, we decided to administer chemoradiotherapy comprising cisplatin and etoposide based on multidisciplinary decision-making. Radiotherapy was performed using a three-dimensional conformal radiation therapy technique. He received 50.4 Gy in 28 fractions with two cycles of cisplatin and etoposide (cisplatin 80 mg/body day 1, etoposide 100 mg/body days 1–3). The patient exhibited grade 2 appetite loss, grade 3 thrombocytopenia, and grade 4 neutropenia during chemoradiotherapy. After chemoradiotherapy, CT revealed shrinkage of the enlarged portal lymph node and an increased CT value around the gall bladder (Fig. 1H). FDG-PET/CT did not reveal increased uptake in these areas 1 year after chemoradiotherapy (Fig. 1i, j). Therefore, we considered a complete response and closely followed up his condition without systemic treatment; we measured tumor markers and performed CT every 4 months. The patient remained healthy with no recurrence for 3 years after chemoradiotherapy.

## Discussion

Chemoradiotherapy has not historically played a major role in the treatment of gallbladder NEC. However, this report indicates that chemoradiotherapy could be an effective treatment option to achieve local control in the management of gallbladder NEC.

Primary NENs can occur anywhere in the body, regardless of the presence of enterochromaffin cells; NEN of the gallbladder comprises 0.5% of the overall NEN incidence and constitutes 2% of gallbladder cancers [2]. NEC is distinguished from neuroendocrine tumors (NETs) by pathological findings of poor differentiation and high-grade tumor. The tumor grade is defined by the mitotic count and Ki-67 labeling index. In the current WHO guideline, NEC is considered a high-grade tumor. Previously, grade 1 and 2 tumors were regarded as NETs and grade 3 neoplasms as NECs [5]. In the intervening years, grade 3 NETs were recognized and shown to be genetically unrelated to NECs. The new classification avoids confusion between these two clinically and molecularly distinct entities [1].

The clinical presentations of most patients with gallbladder NEC are nonspecific, and the most common early manifestation is vague upper abdominal pain [6]. NENs can be classified as functional or non-functional based on the production of peptide substances. Non-functional NENs manifest as symptoms of local disease, such as abdominal pain, weight loss, and jaundice, or symptoms due to metastatic disease. Functional NENs can give rise to symptoms related to the secretion of different peptides in addition to symptoms of local or metastatic disease [7].

It is almost impossible to ascertain the diagnosis of NEC based on imaging findings such as ultrasonography, CT, magnetic resonance imaging (MRI), and PET/CT. It is also impossible to differentiate NEC from other subtypes of gallbladder carcinomas preoperatively. Pathologic examination with immunohistochemical staining is required for a definitive diagnosis of gallbladder NEC. The tumor can stain positive for synaptophysin in 75% of NECs, followed by chromogranin A. In cases of functional NEC, urine 5-hydroxyndoleacetic acid and nuclear imaging studies, such as somatostatin scintigraphy or MIBG, may be useful for diagnosing and evaluating the therapeutic response. Microscopically, NECs appeared as atypical round and oval, small- to intermediate-sized cells with minimal cytoplasm growing in the form of sheets, clusters, or ribbons, often with necrosis and prominent angioinvasion and/or perineural invasion with high mitotic (> 20/10 HPF) and proliferation (Ki-67 > 20%) indices.

Although surgical resection is the only potentially curative therapy for NEC of the gallbladder, most tumors are not surgically resectable. As with other gallbladder carcinomas, NEC can rapidly invade the adjacent liver parenchyma and later cause obstructive jaundice, hindering detection at an early stage. Due to the aggressive tumor behavior and asymptomatic feature at an early stage, patients are often diagnosed at an advanced stage with unresectable and/or metastatic disease, and accordingly have a poor prognosis [8, 9].

Systemic chemotherapy remains the treatment of choice for cases with inoperable or metastasized tumor and can be considered in cases with tumor involvement of the surgical margins. According to a European clinical guideline, cisplatin/etoposide or carboplatin/etoposide is recommended as the standard first-line systemic chemotherapy [10]. In addition, a Japanese clinical guideline recommended platinum-doublet chemotherapy for unresectable and metastatic gastroenteropancreatic (GEP)-NEC [11]. However, there is no established second-line therapy for GEP-NEC. In addition, the role of radiation in GEP-NEC remains unclear. The combination of cisplatin and gemcitabine is a standard chemotherapy for gallbladder carcinoma. However, the efficacy of this regimen was controversial for this case because of the pathological diagnosis as NEC and platinum treatment failure. The role of radiotherapy in the treatment of locally advanced but non-metastatic gallbladder cancer remains unclear. Radiotherapy may be considered in patients with locally controlled disease after first-line chemotherapy [12]. For small cell lung cancer, chemoradiotherapy is recommended for unresectable local disease to improve overall survival and control local progression [13]. The efficacy of chemoradiotherapy for NEC of the gallbladder remains unknown. Few reports have demonstrated that chemoradiotherapy achieved local control in patients with NEC of the gallbladder and prolonged survival (Table 2). According to the literature, one case of NEC of the gallbladder with liver invasion and adjacent liver metastasis achieved a complete response at 12 months after chemoradiotherapy [14].

**Table 2:** Patient and treatment characteristics

Sex	Age (years)	TMN	CTx	Total dose (Gy)	Infield outcome	Recurrence	TTP (months)	OS (months)	Ref
Female	61	T3N2M0	EP	50.4	SD	R	11	15 >	[13]
Male	56	T3N1M0	5-FU	58.4	PR	L + R + DM	5	8	[13]
Male	50	T3N2M0	EP	50/4	CR	No	–	12 >	[13]

*T* tumor, *N* node, *M* metastasis, *CTx* chemotherapy, *EP* etoposide and carboplatin, *5-FU* 5-fluorouracil, *SD* stable disease, *PR* partial response, *CR* complete response, *R* regional recurrence, *L* local recurrence, *DM* distant metastasis, *TTP* time to progression, *OS* overall survival, *Ref* reference

In our case, the patient had already received first-line chemotherapy. However, the evidence for second-line chemotherapy has not been established, and it is very important to control local progression, especially in this case. Therefore, we decided to administer chemoradiotherapy consisting of cisplatin and etoposide according to the regimen for small cell lung carcinoma. The dose of etoposide was low because of the long duration of the previous chemotherapy. However, this case is highly valuable, although there are very few studies on the treatment outcome of chemoradiotherapy for NEC of the gallbladder, which is a rare pathological feature and a very aggressive and lethal disease.

In conclusion, our experience demonstrates that chemoradiotherapy can be used as an effective treatment option for the management of unresectable locally advanced NEC of the gallbladder. NEC of the gallbladder has a poorer prognosis because of its decreased responsiveness to chemotherapy. Chemoradiotherapy may induce a tumor response and achieve tumor control in locally advanced gallbladder NEC. The future role of radiation in combination with chemotherapy or biologic modifiers for the treatment of NEC of the gallbladder remains controversial. We suggest that chemoradiotherapy should be considered part of multimodality therapy for the curative management of advanced NEC of the gallbladder.
